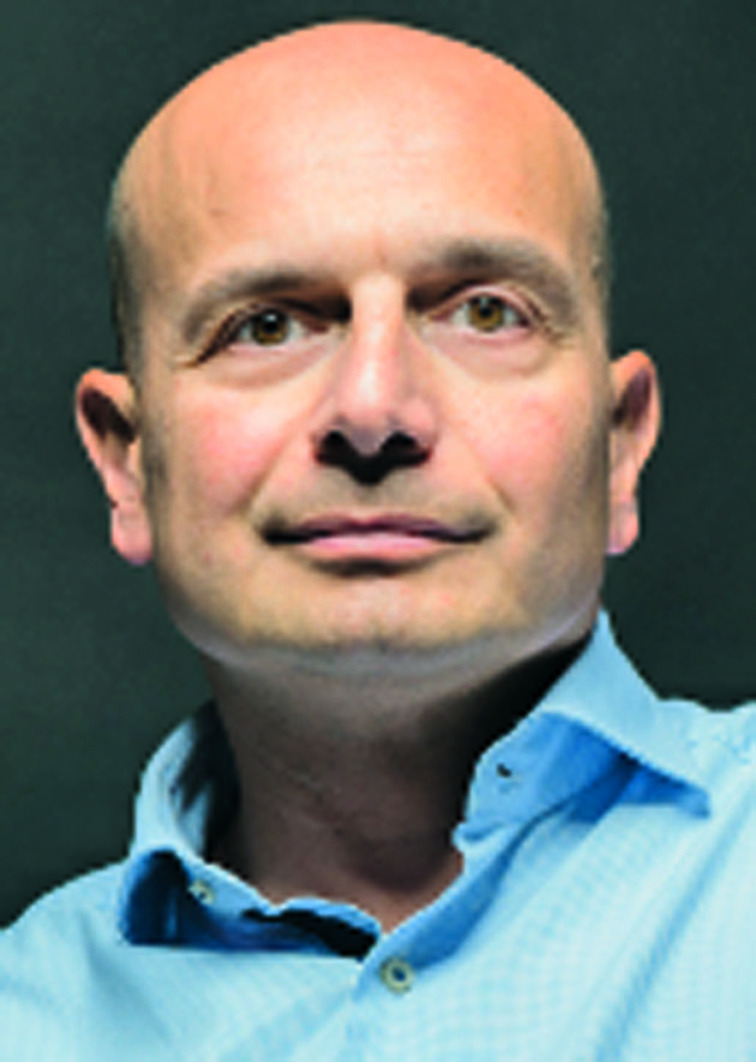# Molecules as Lubricants at the Nanoscale:Tunable Growth of Organic Structures from Nano‐ to Millimeter‐Scale Using Solvent Vapour Annealing

**DOI:** 10.1002/cplu.202400133

**Published:** 2024-10-24

**Authors:** Vasiliki Benekou, Andrea Candini, Andrea Liscio, Vincenzo Palermo

**Affiliations:** ^1^ Institute for Organic Synthesis and Photoreactivity National Research Council of Italy Via Gobetti 101 40129 Bologna Italy; ^2^ Institute for Microelectronics and Microsystems – Rome Unit National Research Council of Italy Via del Fosso del Cavaliere 100 00133 Rome Italy; ^3^ Chalmers University of Technology Horsalsvagen 7B 41258 Gothenburg Sweden

**Keywords:** Organic electronics, Solution processing, Supramolecular chemistry, Thin solid films, Nanofluidics

## Abstract

The creation of ordered structures of molecules assembled from solution onto a substrate is a fundamental technological necessity across various disciplines, spanning from crystallography to organic electronics. However, achieving macroscopic order poses significant challenges, since the process of deposition is inherently impacted by factors like solvent evaporation and dewetting flows, which hinder the formation of well‐organized structures.

Traditional methods like drop casting or spin coating encounter limitations due to the rapid kinetics of solvent evaporation, leading to limited control over final uniformity and order.

In response to these challenges, Solvent Vapour Annealing (SVA) has emerged as a promising solution for realizing ordered molecular structures at scales ranging from nano‐ to milli‐ meters. SVA decouples the self‐assembly stage from the deposition stage by utilizing solvent vapours which can enable rearrangement, movement, and diffusion of large molecules on the surface even on a macroscopic scale. Essentially acting as “molecular lubricants,” solvent vapours enable the formation of well‐ordered molecular films. This review discusses the advancements, obstacles, and promising strategies associated with utilizing SVA for the development of innovative nanostructured thin films, and emphasizes the originality and effectiveness of molecular assembly on substrates achieved through this approach.

## Graphical abstract

Solvent Vapour Annealing (SVA) has emerged as a promising solution for realizing ordered molecular structures at scales ranging from nano‐ to millimeters. SVA decouples the self‐assembly of molecules from the deposition process by utilizing solvent vapors which can enable rearrangement of molecules on the surface, essentially acting as “molecular lubricants.”

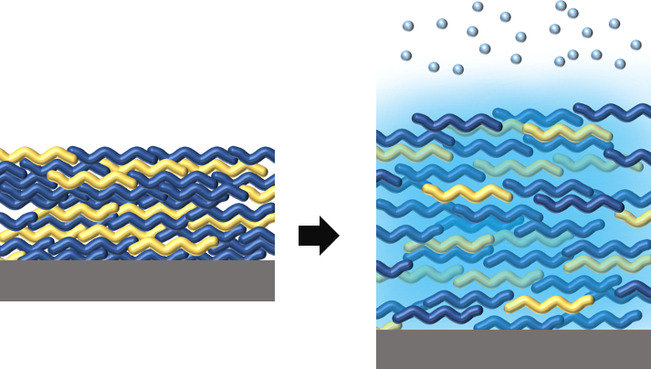

## Introduction

The famous writer and chemist Primo Levi once described the work of chemists with an analogy. “*We are as blind elephants at a watchmaker's table… I say blind, because the things we manipulate are too small to be seen, and so we invented various clever tricks to recognise them without seeing them*.”

Since then, chemists developed even more “clever tricks” to create new molecules and to assemble them, using either covalent or supramolecular chemistry. While the synthesis of molecules takes place mostly in liquids, i. e. 3‐dimensional, bulk solutions, using such molecules in materials and surface science applications requires to assemble them in the form of thin, often nanometric coatings, achieving good uniformity and high order from the nano‐ to the macro‐scale. Uniform, highly ordered layers are required for a broad range of technological applications from protective coatings, to biology to organic electronics.

Despite its apparent simplicity, the process of depositing molecules from a liquid onto a solid involves very complex physics and chemistry, with all the three different phases involved (solid, liquid and gas); control of the final structures can be obtained by understanding and mastering a delicate equilibrium of the interactions between the solute molecule, the solvent, and the target substrate.^[1]^ Molecular order at the nanoscale can be achieved by choosing the right conditions, i. e. the correct solvent, substrate, temperature, and deposition method. Order can be also maximized by slowing the solvent evaporation rate, for example by performing the deposition in a sealed jar, using solvents with low volatility, or working at low temperature. Self‐assembly can also be enhanced by chemical functionalization, for instance side‐chains can be attached to the target molecule to make them able to interact with each other. The process of depositing molecules from solution is, however, intrinsically chaotic, with solvent evaporation, early aggregation in solution, dewetting processes always perturbing the ideal self‐assembly process, as detailed in the following section.

## Challenges in Molecular Deposition Using Conventional Techniques

All the most common techniques to process molecules on surfaces face several disadvantages, in particular for the deposition of delicate organic molecules. Drop casting, spin coating and dip coating all include a step in which macroscopic droplets of solvent should, eventually, be removed from the surface by evaporation – yielding often morphologies dictated by kinetics and fluidics more than by the self‐assembly properties of the molecules, causing poor control and reproducibility.^[2]^

The most common technique to process organic molecules on a substrate is spin coating, with the solution deposited on the sample spinning at hundreds or thousands of rotations per minute. The liquid moves radially outward due to the centrifugal force that is created, and the solution forms a homogeneous layer that becomes thinner when the surplus liquid around the edge of the substrate is removed by sweeping. The non‐volatile components of the suspension, such as the particles, molecules, polymer chains etc. aggregate and deposit on the substrate during this phase. This typically yields a uniform coating on macroscopic scale, but spin coating, being a fast process, does not provide enough time for the molecules to self‐assemble. The final morphology is often due to the kinetics of the solvent evaporation, and rarely corresponds to the most favourable molecular configuration, with the lowest configuration energy.

A slower technique is drop casting. In this approach, a drop of solution is deposited on the substrate, and left there to evaporate. Solvent evaporation increases the local concentration of solute molecules which begin to interact more with each other and with the substrate, leading eventually to ordered nanostructures.

By using drop casting larger crystals or macroscopic structures can grow because of the much slower solvent evaporation process as compared to spin coating.[^3^, ^4^] This is also true for dip coating. Using these solution‐processing techniques results in a film with a degree of uniformity, order and eventually crystallinity that is usually low because physical processes like, for instance, dewetting or drop pinning during evaporation create inhomogeneities on the surface. The most famous (or infamous) example of these defects is due to the “coffee stain” effect.^[5]^

Thus spin coating and drop casting, even if being very simple and widely used, are not ideal techniques to favour a uniform, ordered self‐assembly of molecular systems.

Thermal evaporation in vacuum, on the other side, is arguably the deposition method featuring the best degree of control, leading to molecular films with high quality and uniformity. However, the yield of deposited material is extremely low and in addition it requires very expensive equipment and the use of high temperatures. This often limits the applicability only to fundamental research problems and to molecules that are still stable also in the gas phase and are capable to survive the flight from the evaporation source to the landing on the substrate.

## Solvent Vapour Annealing (SVA)

To overcome the disadvantages mentioned above, one can employ a post‐deposition annealing treatment that effectively decouples the self‐assembly step from the solution processing step.[^5^, ^6^, ^7^, ^8^] Thermal annealing involves the straightforward addition of thermal energy to the system, enabling it to achieve a more stable thermodynamical equilibrium. Although these processes are widely employed in the fields of ceramics and metals, their benefits in organic materials are limited, due to the temperature limits at which molecules can be subjected and the restricted molecular diffusion that can occur on a substrate. In Solvent vapour annealing (SVA), instead, solvent molecules can adsorb on the sample, condensed from vapours. These solvent molecules surround the deposited organic molecules, acting as small molecular lubricants prompting the rearrangement, movement, and diffusion of the molecules on the substrate, even on a macroscopic scale.

The standard SVA process consists of three key steps.^[9]^

1) Initially, target molecules are deposited from solution on the target substrate using any of the above‐mentioned standard deposition techniques such as spin coating, drop casting etc. The solvent used in this stage should be good at solubilizing the molecules but does not need to favour molecule self‐assembly. This preliminary deposition is needed to place on the substrate the molecules that will undergo self‐assembly in the successive SVA treatment.

2) The prepared thin film is inserted in a sealed chamber, where a reservoir of a solvent is present. Solvent evaporates within the chamber saturating the atmosphere and condensing on the sample. Such solvent can be the same used for the deposition, or a different one. This solvent can be even a mediocre solvent for the molecules, because the goal is not to re‐dissolve the deposited layer, but only to change its structure. In the right conditions, the vapour will condense in a thin liquid layer on the substrate, which can reach a thickness spanning from ≈10–200 nm,^[10]^ covering the whole sample. Such thin layer of liquid can be described as a quasi‐2‐dimensional solution and can render the molecules mobile with rather high lateral diffusion, while keeping them constrained in close proximity to the surface. If the amount of solvent deposited is low compared to the solute and/or if the molecules are large and entangled with each other (as for example, in polymer coatings) the solvent‐molecule system is better described as a swollen material rather than as a classic liquid solution.

At difference with other deposition techniques which try to achieve maximum order during the deposition, the ideal starting substrate for SVA is one featuring nano‐crystalline or even amorphous coatings. This is because in a thin layer solution as the one established during SVA, molecules shall undergo a dynamic, reversible crystallization‐dissolution forming a two‐ phase mixture solid‐liquid equilibrium. If the solid is formed by tiny aggregates with large surface area, the mixture is far from thermodynamic equilibrium and can approach equilibrium by an increase in the size of the solid phase, minimizing the total interfacial area, a process often termed *Ostwald ripening*.

Assuming that the small crystals and large aggregates have the same crystal structure and thus chemical potential μ, the driving force for the ripening process is the well‐known curvature dependence of the chemical potential on local curvature:

(1) $$\mu =\mu_{0}+V_{m}\cdot \gamma \cdot k$$

where *k* is the mean interfacial curvature and *μ_0_* is the chemical potential of an atom at a flat interface, *V_m_* is the molar volume and *γ* is the surface energy.^[11]^ This means that atoms will flow from regions of high to low curvature, small crystals will dissolve in the solution layer, and large crystals will grow. The higher the curvature of the initial aggregates, the stronger the driving force. If the starting aggregates have a different structure from the large crystals with higher chemical potential (i. e. they are amorphous), the SVA will be even more effective.

Upon interaction with the solvent molecules, the material structure can change and rearrange. This rearrangement can vary from small reorganization and crystallization on the nanometer scale, to long‐range diffusion and creation of structure on the millimeters scale.

3) Eventually, the solvent vapour is allowed to evaporate, leaving behind the reorganized structure.

**Figure 1:**
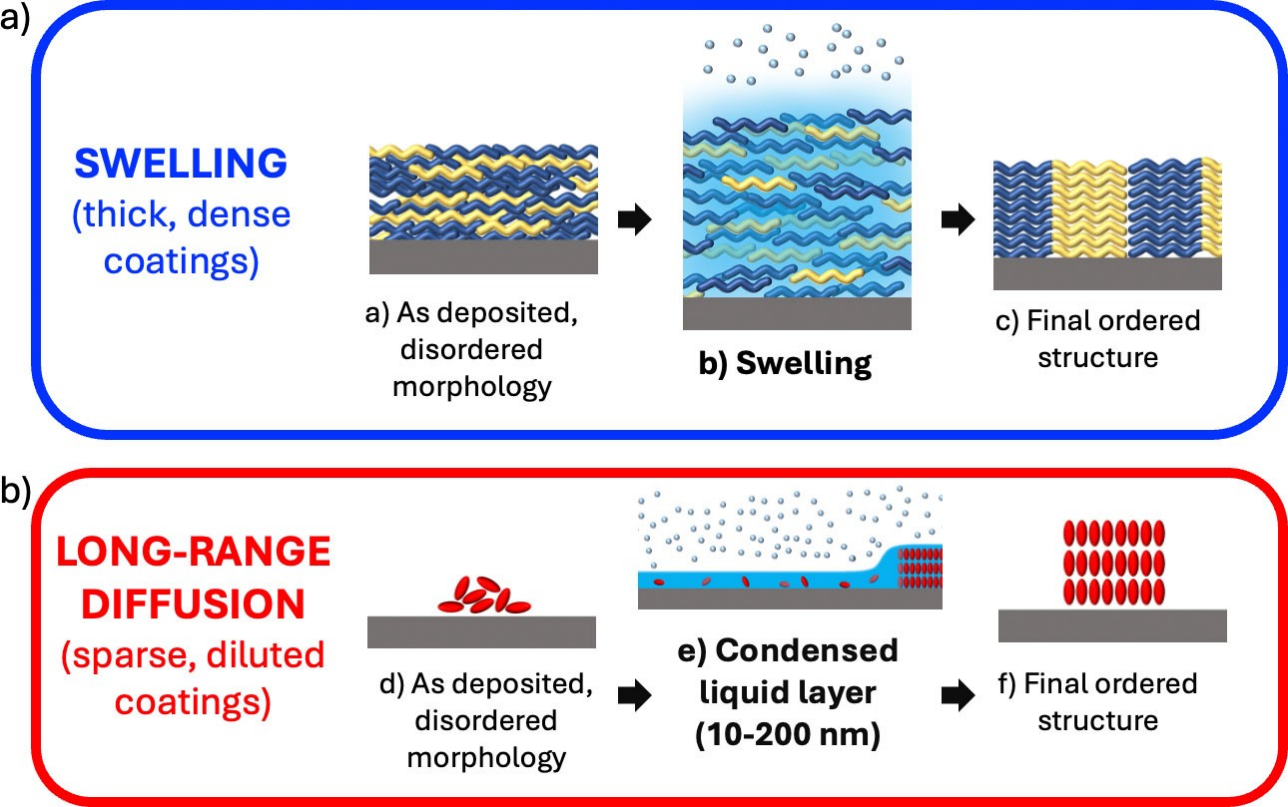
Scheme of the most common types of SVA. a) SVA of thick coatings, as example polymer layers, that can swell upon solvent condensation. b) SVA of sparse coatings, as example small molecules, able to dissolve in the condensed layer and diffuse to assemble in large crystals.

The most common SVA treatments can be classified in two major categories, depending on the state of the initial material. SVA is often performed on a thick, continuous layer (Figure 1a), typically a polymer layer hundreds of nm thick. In this case, the solvent will penetrate in the coating swelling it. The molecules will be able to move and rearrange in the swollen material, looking for the most favourable arrangement. In this case, the changes take place only on local scale; condensation of bulk liquid on top of the film should be avoided, because it will cause polymer dissolution and film dewetting. SVA has been typically used in this way to create periodic patterns of block copolymers.^[12]^ This case will be treated in detail in Section 4.

If, instead, SVA is performed on discontinuous, low‐density coatings (Figure 1b), a thin layer of liquid solvent (≈10–200 nm) will form by condensation on the substrate. The molecules can dissolve in the condensed liquid layer and, thanks to this extensive freedom of movement, diffuse even on large distances,^[6]^ like in a conventional solution; in the right conditions large structures can be formed with a change in the surface morphology that can be observed even with naked eyes. This case will be treated in detail in Section 5.

The condensation of the liquid layer can be controlled by varying the temperature of the substrate with respect to a solvent reservoir, in a process termed temperature‐enhanced solvent vapour annealing (TESVA).^[5]^ Since the first demonstrations of this technique in 2010,^[13]^ much progress has been achieved, and different original approaches have been developed to exploit SVA and techniques derived from SVA to pilot molecular assembling and obtain novel nanostructured coatings.

In this review, we will describe some of the results obtained in this field. The goal of this review is not to be comprehensive, given the several hundreds of publications dealing with SVA, but to focus on the works which, in our opinion, used original and promising approaches, architectures or setups to solubilize molecules on a substrate, and arrange them in a (more) ordered fashion.

## SVA for Self‐Assembly in Dense Polymer Layers

**Figure 2:**
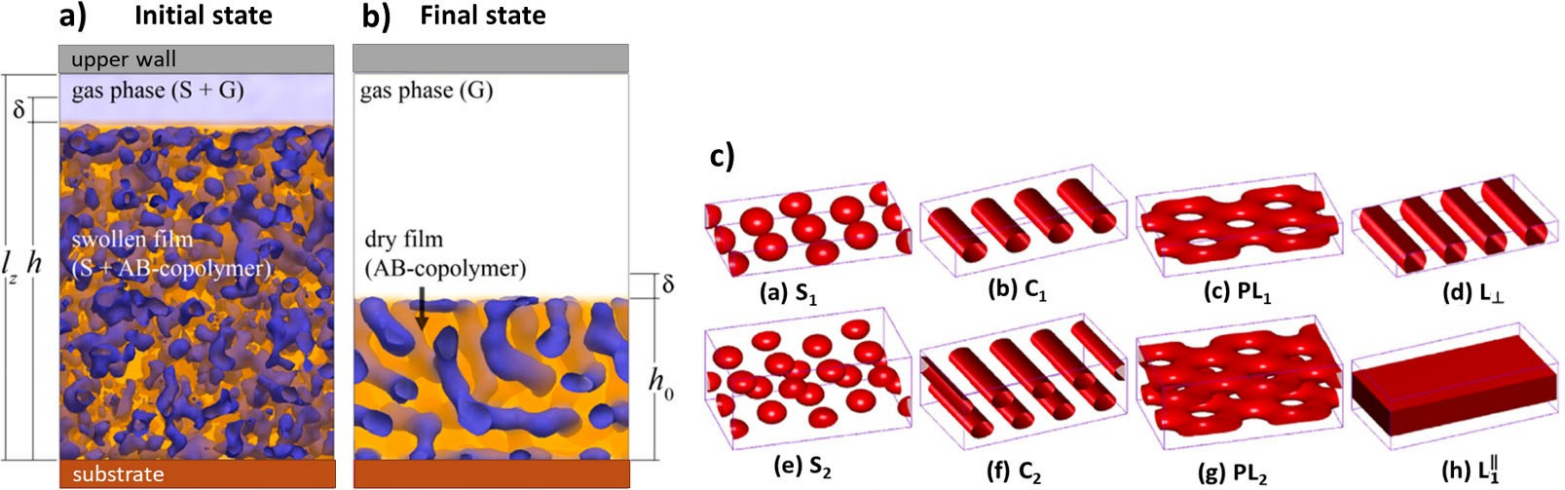
Simulation of the swelling of a block copolymer a) during and b) after SVA. Adapted from Ref. [15]. c) Some examples of possible BCP architectures attainable by phase separation and SVA. Adapted from Ref. [16].

**Figure 3:**
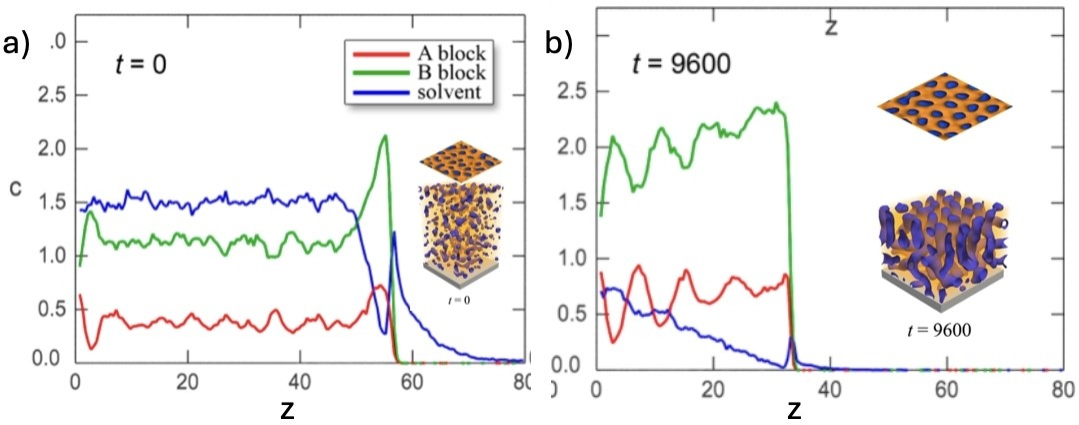
a) Distribution of solvent in the swollen A–B block copolymers during SVA, as obtained by Dissipative Particle Dynamics simulations; z is the vertical distance from the substrate, t is the dimensionless time. b) Distribution calculated after solvent evaporation, showing partial removal of solvent and periodic oscillations in the abundance of A and B copolymers. The insets show the internal structure of the film and topography of the free surface. Adapted from Ref. [15].

SVA has been intensively used to modify and improve the morphology of dense, continuous polymer layers. In particular, this technique has been very successful to enhance phase separation and order of block copolymers (BCP), stimulating their self‐organization of nanometric patterns like nanosphere arrays, alternating lamellae, cylinders patterns or irregular inter‐digitated networks (Figure 2 and 3).^[14]^

This work was stimulated initially by the idea of using such self‐assembled patterns as templates for lithography on a scale length of ≈10 nm. SVA modifies the polymer layers due to increased mobility of the polymer blocks caused by swelling, microphase separation and reduction of surface tension caused by the solvent vapours. SVA lowers the system's free energy, facilitating conformational changes and the development of organized lamellar domains.^[17]^ The driving cause is the interfacial tension between the microdomains A and B, which in turn depends on Flory–Huggins segment‐segment interaction parameter χ_AB_ between the two blocks.^[16]^

**Figure 4:**
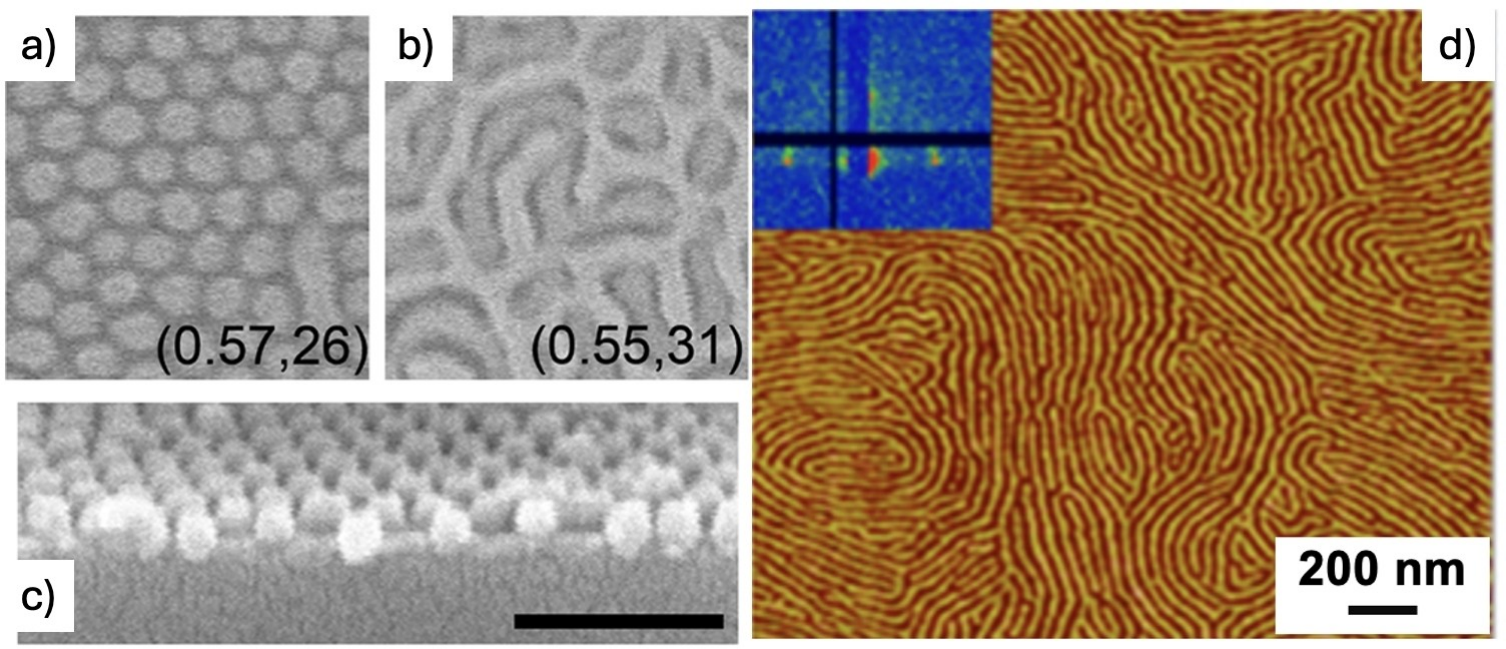
a and b) Periodic patterns of PS‐PDMS block copolymers formed by different SVA in toluene. Each image reports the fraction of toluene in vapor and total vapor pressure (Torr). c) Cross‐section of vertical cylinder formation. From Ref. [21]. d) AFM image of PS−P4VP copolymers after SVA in chloroform. The inset shows the corresponding GISAXS map. From Ref. [20].

Solvent annealing has demonstrated to be an ideal technique to modify the structure of such films, and has been performed on a variety of copolymers like poly(styrene‐butadiene), poly(styrene‐methyl methacrylate), poly(styrene‐2‐vinylpyridine), poly(styrene‐isoprene‐styrene), poly(styrene‐ dimethylsiloxane) poly(styrene‐butadien‐styrene) and poly(styrene‐4‐vinylpyridine) as well as mixtures block‐copolymers and the respective homopolymers of poly(styrene‐methyl methacrylate) (Figure 4).[^10^, ^12^, ^14^, ^16^, ^18^, ^19^, ^20^, ^21^, ^22^, ^23^] We will give some examples of SVA application in this field; please note that this is just a small selection of all the results published in the recent years on the SVA of polymers.

In 2023, Babutan et al.[^17^, ^24^] employed solvent vapour annealing to promote the self‐assembly of different block copolymers (BCPs) into ordered nanostructures. To this aim they utilized a homemade setup that consisted of a shallow sample chamber that quasi confined the solvent and the polymer molecules. Using a nitrogen‐based solvent “bubbling” system, precise volumes of solvent vapours were introduced into the chamber to cause the swelling of thin BCP coatings. The process was observed in situ through an optical microscope fixed above the set up, and the quality of the results was assessed via AFM. In a similar way, the same group achieved the crystallization of Poly(ethylene oxide)‐based triblock copolymers by SVA.^[25]^

More recently, in 2024, Doerk et al.^[19]^ achieved much greater variability and control on final morphology by overcoming the limitations due to the relative length of the two polymer branches. To this aim, they performed SVA on symmetric PS‐PMMA block copolymers mixed with their respective homopolymers. SVA performed with THF, a solvent weakly selective for PS, promoted assembly of metastable lamellae, which could be further stabilized by thermal annealing. SVA with a strongly selective solvent like acetone yielded instead morphologies depending solely on the total polymer blend composition.

## SVA for Self‐Assembly of Thin Films of Small Molecules

**Figure 5:**
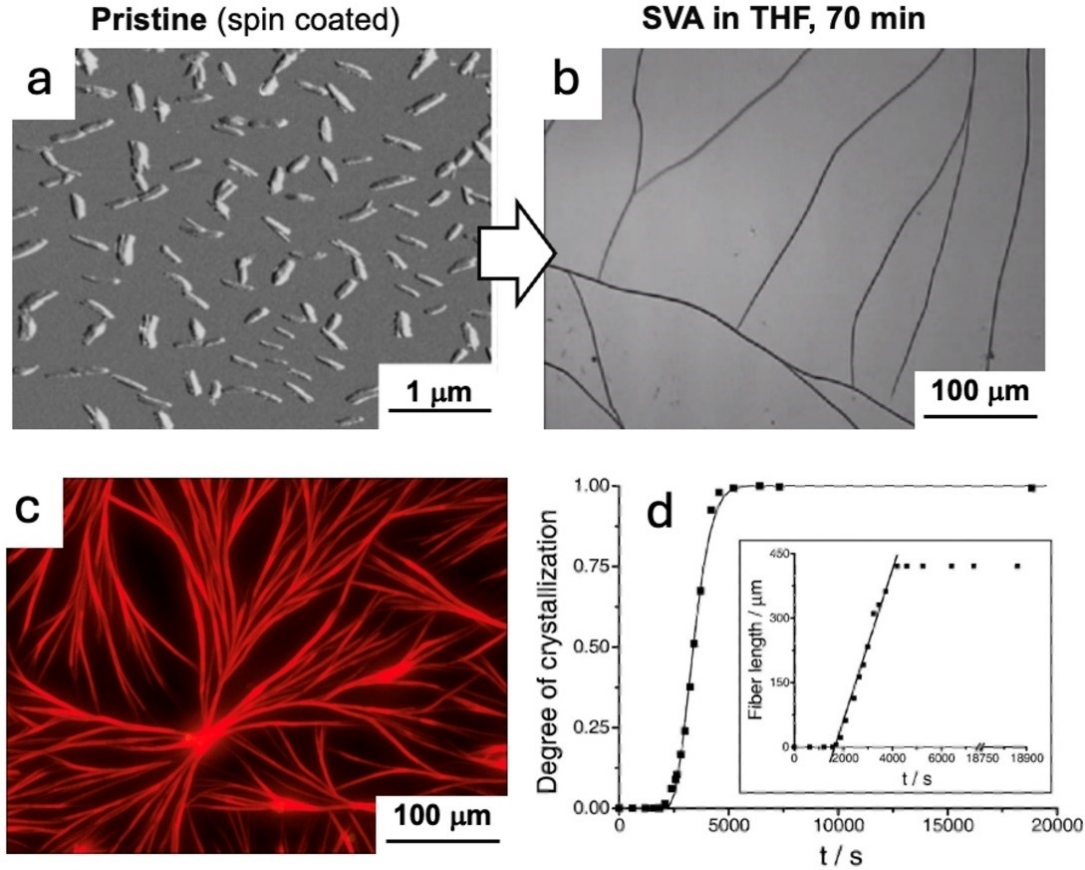
a) Atomic force microscopy (AFM) images nanostructures of perylene molecules before SVA. b) Optical microscopy image of macroscopic perylene fibers grown on the same sample after SVA in THF. c) Fluorescence image of the fibers, showing their red emission.^[6]^ d) Kinetic profile of the fibers’ growth. The solid line represents the fit of the Avrami equation to the kinetic data, whereas the dots represent the degree of crystallization versus time. A single fiber's length versus time is shown in the inset; the linear region's slope is (183±7) nm s^−1^. Figure adapted from Ref. [6].

If SVA is performed on a flat solid substrate like, e. g., silicon, covered by a sparse array of molecules, a truly liquid layer will condense on the substrate, solubilizing the molecules present on the surface. Some of the most spectacular outcomes of SVA have been achieved with small molecules that, thanks to the fast diffusion in the condensed liquid layer, can perform reversible self‐assembling going from small disperse nano‐aggregates to macroscopic structures visible by eye. As example, the well‐known organic semiconductor perylene diimide (PDI) has been used to create millimeter‐long crystalline fibers with a sub‐micrometer cross‐section on various solid substrates (SiO_x_, glass, graphite, and mica).^[6]^ Initially, the PDI was deposited on these different substrates by spin coating from tetrahydrofuran (THF), obtaining a disordered morphology featuring small, needle‐like structures with typical lengths below 350 nm (Figure 5a).

Upon exposure to the vapours of THF, the needles underwent re‐organization, forming fibers with length up to few millimeters, a remarkably high aspect ratio exceeding 10^3^ and uniform length and thickness, typically in the range of 200 nm (Figure 5b and c).

Given the macroscopic scale of this process, its kinetics could be monitored easily using an optical microscope equipped with a long‐focus objective to visualize the sample while into the SVA chamber. Employing SVA, the growth of macroscopic fibers on SiO_x_, glass, graphite, and mica was demonstrated.^[6]^ Interestingly, the growth continued also over the substrate's edges, indicating that the drive toward fiber formation is significantly strong. According to preliminary findings, it was possible to achieve surface‐oriented fiber growth on mica, which permitted directionality control and results in a parallel orientation of the fibers on this substrate The growth direction of the fibers on graphite was also influenced by the crystal nature of the substrate, however, in this case shorter needles were formed. This evidence could be explained by the apolar nature of graphite, which promotes stronger surface‐molecule interactions that hinder the formation of long fibers and the self‐assembly of PDI monomers. Moreover, the same approach was demonstrated also on the top of gold contacts on silicon, that are suitable for electrical characterization. Remarkably, compared to previous works showing ordered PDI nano‐structures, this SVA method yielded an unprecedented long‐range order, extending up to the millimeter scale, with a high degree of organization and alignment in the formed fibers.

The self‐assembly process was reversible, as demonstrated through a series of assembly and disassembly steps cycles between SVA of THF and CHCl_3_, highlighting the dynamic nature of the self‐assembly process.

The process followed a nucleation‐governed growth mechanism, reminiscent of an Avrami‐type mechanism, which could be fitted as:

(2) $$Y=1-e^{-{\left[k\left(t-t_{i}\right)\right]}^{n}}$$

where *Y* is the degree of crystallization, *k* is the crystallization rate constant, *t_i_* corresponds to an induction period preceding the growth and *n* is the Avrami exponent. Experimental kinetic growth data could be fitted well using n=2, indicating the presence of homogeneous crystallization characterized by continuous nucleation and growth (Figure 5d).

These were not the first studies using SVA to improve the properties of a material, and previous publications already used it to improve the crystallinity of bulk polymers, or the electronic properties of organic semiconductors (see[^6^, ^13^] and references therein). In such works, however, the increase in crystalline character was due to reorganization on the nanometric scale, rather than molecular transport over macroscopic distances, with the mesoscopic structure of the surface remaining unchanged. Conversely, quantitative analysis of the fibers’ growth process demonstrated the presence of long‐range mass transport during the self‐assembly process. The molecular motion of PDI was found to occur over at least hundreds of micrometers, indicating efficient and extensive transport of the PDI molecules in the thin solvent layer.^[6]^ Upon SVA, diffusion could be estimated to be D≈10^−13^ m^2^ s^−1^, hundred times faster than what can be achieved in vacuum (D≈10^−15^ m^2^ s^−1^), even if much slower than what observed in bulk liquids (typically D≈10^−9^ m^2^ s^−1^) or in gas (D≈10^−5^ m^2^ s^−1^).

Chemical functionalization of the molecules can be combined with SVA in different solvents to achieve even more versatility in assembling structures such as fibers, domes or needles.^[7]^ Upon SVA in THF, some PDI derivatives exposing branched side alkyl chains formed hierarchically‐organized spherical agglomerates, containing domains of aligned nanocrystals having a high aspect ratio. By performing the same SVA on PDI derivatives with longer side chains, instead, mesoscopic structures with highly crystalline, zig‐zag columnar packing were obtained. Upon SVA in THF, Hexabenzocoronene (HBC) molecules could be rearranged from a nanometric network having a height of ≈2 nm to needles with length <1500 nm, even if THF was not the best solvent for this molecule. By performing SVA in chloroform, a better solvent, large tapered needles with lengths >5000 nm and width ≈500 nm were obtained. The needles showed a hierarchical structure, formed by smaller needle‐like aggregates.

Since PDI is an electron acceptor and HBC and electron donor, their blends are of interest for photovoltaic and electronic applications.^[8]^ SVA was therefore applied to PDI/HBC blends in order to improve their performances. In the case of the blend, the self‐assembly behaviour was different from the one observed in either pure PDI or HBC. By tuning the experimental conditions and the molecular structures, SVA could yield uniform acceptor/donor blends, with no evidence of phase separation at the microscopic scale, or, conversely, the two molecules could self‐assemble each one in its own structures, i. e. PDI in long slender fibers as mentioned above and HBC in small and thick crystals.^[8]^

**Figure 6:**
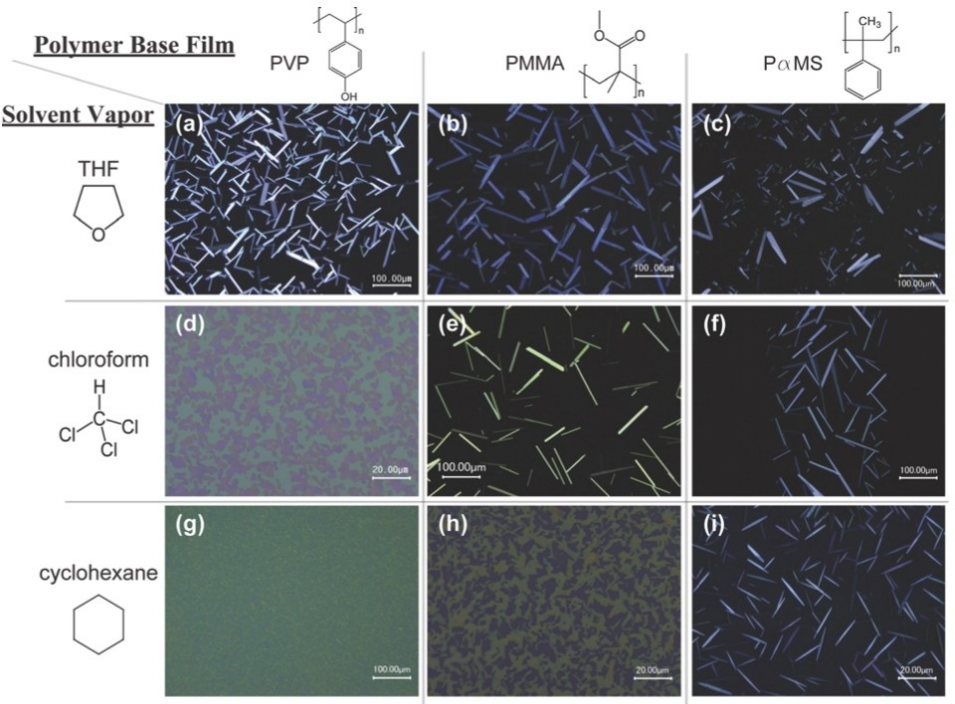
Polarized microscope images of vacuum sublimed films of a benzothiophene derivative (C8‐BTBT) after SVA of 15 hours, with different solvent vapours, on different polymer substrates. From Ref. [26].

In most of the published works SVA is performed either on a highly permeable polymer layer able to swell (Figure 1a) or on a flat, non‐reactive substrate, where the condensed vapours would form a liquid layer (Figure 1b). In 2012, Liu et al.^[26]^ combined these two different approaches, performing SVA on small molecules deposited on a polymer layer which was able to influence the growth of the semiconductor. They managed to grow large crystals of different organic semiconductors by SVA on a thin layer of PMMA (Figure 6). Crystal quality in presence of the polymer layer was much better than what obtained on bare hydrophilic silicon. It was also better than what obtained on silicon coated with Hexamethyldisilazane (HMDS), which has a hydrophobicity comparable to PMMA, so the improvement could not be explained by a simple change in surface energy of the substrate.

There was no correlation between crystal quality and the vapour pressures or polarity index of the solvents used for SVA. Instead, the authors found that the formation of crystals was strongly related to the miscibility of polymer substrate with the solvents used for SVA.

Optical microscopy showed that the initial disordered film of molecules dissolved upon SVA on PMMA within 1 min, while the same film on a bare Si/SiO_2_ substrate did not show any dissolution even after 30 min. According to the authors, the presence of soluble PMMA dramatically enhanced the total uptake of chloroform and the absorbed solvent significantly increased the molecular mobility and thus the diffusion rate. The organic crystals could then grow via Ostwald ripening. The uptake of solvent by the PMMA substrate was demonstrated by formation of protruding polymer features around the organic crystals, probably due to PMMA swelling during the SVA process and climbing up the crystal edges due to capillary force. If PMMA was too thick, solvent uptake became so significant that generated a bulk liquid layer thick enough to float all the crystals to the sample edge. In a similar approach, Zhu et al. used a PMMA substrate and a slight (5°) tilt of the sample to increase the fluidity of the anthracene molecules during SVA and to guide their growth direction to form microwire arrays.^[27]^

SVA can be used to influence the self‐assembly not only of small single molecules but also of larger building blocks. In 2018, SVA was used to promote the aggregation of large spherical structures called IBAN (intensely and broadly absorbing nanoparticles), due to their interesting optical properties. Each IBAN was a large spherical object formed by 44 silver atoms coated by a monolayer of 30 thiol ligands, and SVA was used to promote their aggregation into either 2‐D layered materials or 3‐D crystals.^[28]^

To this aim, a solution of IBAN in acetone was deposited on silicon, yielding an amorphous layer, easy to transform into more ordered structures. Then, SVA in acetone favoured the growth of large 3‐D crystals of pristine IBAN similarly to classic molecular crystals, assembled thanks to weak, reversible supramolecular forces. The IBAN structure was maintained also in the crystals, and they could be re‐dispersed in solution to re‐obtain individual IBAN particles. Conversely, SVA in dichloromethane (DCM) caused all IBAN particles to undergo a structural and chemical change, forming 2D layered structures of oxidized silver atoms, bound together to form a continuous covalent structure protected on the upper and lower sides by the thiol molecules. Such covalent 2D structures showed a better order and thermal stability than the 3D supramolecular crystals and had the shape of elongated needles. This dramatic but ordered change of structure at the nanoscale could be followed in its different stages using X‐Rays Diffraction (XRD), Atomic Force Microscopy (AFM) Grazing‐incidence wide‐angle X‐ray scattering (GIWAXS) and Grazing‐incidence small‐angle X‐ray scattering (GISAXS).

## SVA Mechanism: Fluid Dynamics at the Nanoscale

The performance of SVA will of course depend on how effectively the solvent selected will wet the surface and solubilize the target molecules. The most effective solvent will not always be the best solution, given that it could screen inter‐molecular interactions, and thus self‐assembly.^[1]^ Ideal solvents will be able to mobilize the molecules on the surface, or polymers into the polymer layer, allowing them to interact and self‐assemble in ordered structures. A straightforward way to select the best solvent is the Hildebrand solubility parameter δ, which is related to the cohesive energy density of a material. As example, selective SVA has been successfully performed on polyisoprene (δ≈16.6 MPa^1/2^) and polystyrene (δ≈18.4 MPa^1/2^) using solvents like n‐hexane (δ≈14.9 MPa^1/2^), which dissolves better polyisoprene, and THF (δ≈19.4 MPa^1/2^), which dissolves slightly better polystyrene.^[22]^ A more refined, less deterministic approach was used by Hendeniya et al.^[20]^ using Flory−Huggins interaction parameters χ and adsorption‐desorption solvent vapour isotherms to understand swelling behaviour and govern the morphological evolution. This allowed to study in detail the swelling behaviour and the morphological evolution of the styrene‐(4‐vinylpyridine) block copolymers and select the best annealing pathways for grain coarsening while preventing macroscopic film dewetting under SVA (Figure 6d).^[20]^

**Figure 7:**
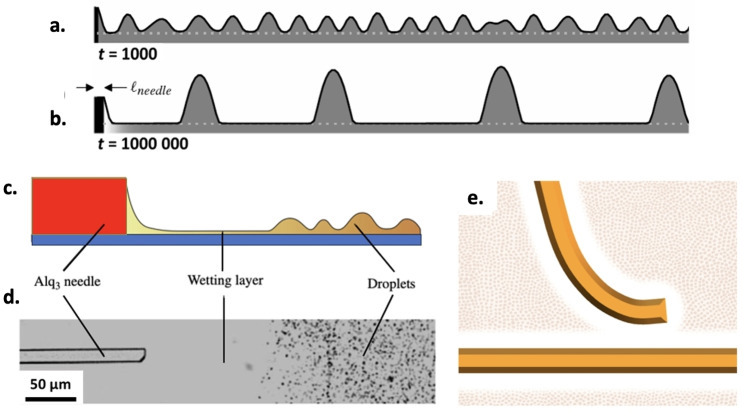
a and b) Cartoon depicting evolution of a thin condensed film during SVA, adapted from Ref. [29]. Initially, a uniform liquid layer covers the sample (not shown). Then, the film becomes unstable and breaks down into small drops. In b) the drops collapse and combine to form larger, more coarsely‐spaced drops at late times. The ultra‐thin‐film thickness at equilibrium is indicated by the grey, dotted line. c) Scheme of a needle growing into a fluid film, with needle height much larger than the condensed drops. d) An optical micrograph of a typical Alq_3_ needle after 3 hours of SVA. The rectangular needle (entering from the left of the image) is surrounded by a clear wetting layer of fluid followed by a region characterized by small droplets. It should be noted that although the micrograph displays a top‐down view of the substrate, the scheme in c) is drawn in side view. Adapted from Ref. [29]. e) Cartoons representing the repulsive interaction and bending of a fiber approaching a pre‐existing one. Adapted from Ref. [6].

In SVA on dilutes systems, The solvation of the target molecules in the condensed solvent layer will drive self‐assembly, creating complex nano‐fluidic dynamics. In 2013, Yu et al.^[29]^ presented a theoretical model describing how thin crystalline fibers grow when the starting materials is exposed to SVA. The process was modelled as a solidification from thin‐film liquid mixtures, and studied combining theoretical arguments, numerical simulations and experimental measurements on the growth of (tris‐(8‐hydroxyquinoline) aluminium) (Alq_3_) upon SVA in methanol. The total fluid pressure in the liquid at time *T* and along horizontal coordinate *X* can be modelled as:

(3) $$P\left(X,T\right)=\;-\sigma \;\frac{\delta^{2}h}{\delta h^{2}}\;+\;\Pi \;\left(H\right)$$

where the first term is related to the local curvature of the film, with *σ* being the surface tension and *h* the height of the liquid layer. The second term is the so called “disjoining pressure”, due to intermolecular interactions between liquid, substrate and vapour, which are relevant for films with *h*<100 nm. The evolution of the thin wetting layer formed upon SVA was modelled by a lubrication equation and an advection–diffusion equation. In the liquid layer, the competition between intermolecular forces and surface tension drives the formation of drops connected by a thin wetting film (Figure 7a and b)

In contrast to what occurs in macroscopic systems, the solvent evaporation from the droplets pushes the fluid through the ultra‐thin film. In this way individual drops, rather than merging and coarsening through motion, grow along a single dimension creating extremely long needle‐like structures (Figure 7c and d). Fluid flows to the needle due to the pressure difference between the meniscus and the drop closest to the needle tip, i. e. the first drop closer to the needle (Figure 7c). When the needle height is larger than the typical drop height, then the pressure in the meniscus will be lower than the pressure in the film; this is because the meniscus at the needle has a positive curvature and creates a low‐pressure region, while the negative curvature of the drop closest to the needle tip creates a high‐pressure region.

The model reproduces analytically the power‐law behaviour of the needle growth observed experimentally as: *L_needle_ ∼ t* ^*γ*^, where *t* is time from nucleation, and *γ* is the so‐called “growth exponent”. The γ values range between 0.29 and 0.5 due to the interplay between coarsening, diffusion, and needle height. The model considers only purely one‐dimensional growth (length only) and in the case of contributions from other dimensions ‐ variation of the height and width of the needles ‐ the authors suggest that the value of gamma lies in the range 0.5 < γ < 1.

Noteworthy, the same model can describe well also other systems, such as a benzothiophene derivative (C8‐BTBT) in THF,[^26^, ^30^, ^31^] HBC derivate in CHCl_3_ ^[5]^ and PDI in THF^[6]^ (Table 1). We stress in particular the observation reported in ref.^[6]^ where the PDI fibers reach lengths of up to one centimeter, which to the best of our knowledge is the maximum length reported in the literature for similar systems. This system showed apparent repulsive, interactions between growing PDI fibers, which coiled or deviated to avoid each other (Figure 7e). Although other mechanisms may also be involved in growth, the model developed by Yu at al. helped to rationalize this repulsive interaction between growing fibers, which was initially interpreted as due to depletion of PDI in an otherwise uniform liquid layer, or as the presence of liquid crystalline layer of oriented molecules. In view of the results then obtained in ref.,^[29]^ this apparent repulsion could instead be attributed to the above‐mentioned pressure difference which drives flow toward the needle tip and promotes the collapse of drops near the tip, creating a smooth area around each needle, depleted of molecules, thus hindering the growth of competing fibers in the same area.

**Table 1:** Power‐law behaviour of crystal growth in different published works.

System	Growth exponent	Notes	Ref.
Alq_3_ in methanol	0.29<γ<0.5	System from which the theoretical model was developed	[29]
C8‐BTBT in THF	γ=0.45	Digitalized data from Figure 2 of the work.	[30]
	γ=0.37	The article takes into account also the contribution of the substrate, not considered in the model.	[26]
	γ (volume)≈1 γ (length)≈0.6	This work takes into account that needle growth is not purely 1D as the thickness and width also vary over time. Also, the volume growth shows a power‐law behaviour. The γ value for length is calculated subtracting the trend of width and heights from the contribution of the volume.	[31]
HBC derivate in CHCl_3_	γ≈0.81	In this case the power‐law behavior is also observed in non‐needle‐like structures. Growth is not purely 1D.	[5]
PDI in THF	γ≈1	This is the case known to us with the longer needle length than those reported in the literature.	[6]

## Temperature‐Enhanced Solvent Vapour Annealing (TESVA)

SVA is often used to improve the quality of some materials but provides no precise control on the amount of solvent interacting with the target molecules. A more powerful and versatile version of this technique is temperature‐enhanced solvent vapour annealing (TESVA), where the temperature of the sample and of a solvent reservoir can be controlled independently, in this way either amplifying or inhibiting the effects of SVA.^[5]^ By controlling temperature, TESVA allows to condense a thin solvent layer on the substrate, to promote molecular migration in the deposited material. This is typically achieved using few °C of temperature difference, in contrast to thermal annealing, which increases the mobility of molecules at a surface by raising the substrate temperature to significant levels. Therefore, when the substrate is cooled with respect to the reservoir (ΔT<0 °C), TESVA can yield rearrangement on extremely large scale, and the molecules self‐assemble in new macroscopic structures visible by naked eye.

**Figure 8:**
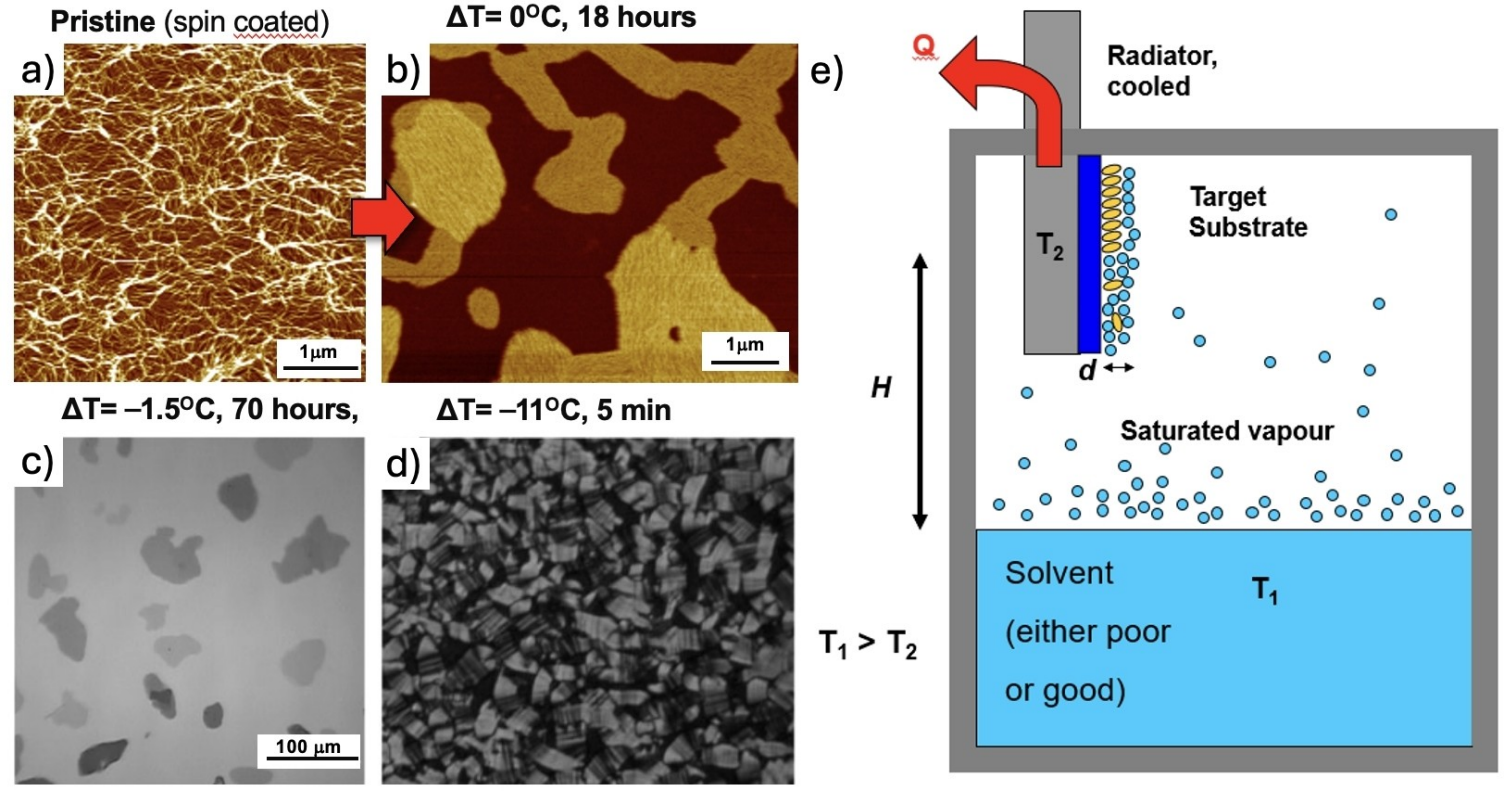
a) AFM of modified HBC molecules deposited on silicon. b) Same sample after 18‐hour at *ΔT*=0 °C. c) Optical Microscopy image of HBC islands grown on SiO_x_ after 70 h of TESVA at *ΔT*=−1.5 °C. d) A thick layer of crystals observed under crossed polarizers obtained via TESVA for 5 min at *ΔT*=−11 °C. e) Schematic illustration of the temperature‐enhanced solvent vapour annealing (TESVA) process and instrumentation. Figure adapted from ref..^[5]^

Figure 8 illustrates schematically the first TESVA setup. The TESVA process begins with the traditional spin coating, in which a solution containing the target molecules is firstly deposited on a flat surface, typically a SiO_x_ substrate. After that, the sample is placed inside a sealed annealing chamber that is filled with a volatile solvent's vapours as in normal SVA. The primary parameter, the difference (ΔT) between the substrate temperature T_sub_ and the liquid solvent reservoir temperature T_solv_, is precisely controlled using a Peltier module. Only a few nanometers thick layer of liquid is adsorbed on the surface in the case of positive or null ΔT, while macroscopic solvent condensation can be achieved at significantly negative values of ΔT. In between these extreme cases, the amount of the solvent condensed on the sample can be tuned by tuning ΔT. As a test model, TESVA was used to assemble modified HBC molecules, featuring alternated hydrophilic (i. e., triethylene‐glycol) and hydrophobic (i. e., dodecyl) side chains, to foster phase separation at the nanoscale. Due to the presence of an aromatic core, hydrophilic and hydrophobic moieties, such molecules are an interesting test system for TESVA.

The initial coverage was quite disordered on nanometric scale, with HBC arranged in short fiber‐like structures (Figure 8a). Upon prolonged SVA in CHCl_3_, the isolated fibrils adsorbed on the neat substrate disappeared and all the material assembled into micrometric islands (Figure 8b). The final morphology showed discrete islands surrounded by wide areas of uncoated substrate, where no molecules could be observed by AFM, providing unambiguous evidence for the diffusive behavior of the molecules on nanometric scale. Island growth was a dynamical and reversible process, as demonstrated by the presence of island ripening. At ΔT=0 °C, though, material reorganization was confined to the micrometer scale, even for long annealing times, and no long‐range transport of material took place.

Performing TESVA with a negative but still relatively small ΔT=−1.5 °C, no macroscopic solvent condensation was observed, but the optical microscopy image showed islands with a lateral size of several tens of micrometers (Figure 8c). By using more negative values of ΔT, macroscopic droplets of solvent could be observed on the substrate, allowing to crystallize even thick layers of molecules, approaching the conditions previously described for SVA swelling of polymer layers (Figure 8d).

It is noteworthy that in TESVA even non‐solvents, that is liquids that usually are not able to solubilize the molecule, can modify the adsorbate morphology; a highly polar solvent such as methanol, which was a non‐solvent for our HBC, could cause a change in morphology and local re‐organization in larger bundles of fibers.

SVA could be used to assemble structures not only through supramolecular assembly of a single type of molecule, but also by combining different species such as polymers or small ions to form cross‐linked structures. In 2017, Lee at al. used SVA to produce controlled molecular‐scale polyaniline (PANI) polymer chains, cross‐linked by metal ions.^[32]^ For this, solution mixtures of PANI‐and metal salts were spin‐coated or drop‐casted on glass, then annealed in dimethyl‐formamide (DMF). Being DMF a high‐boiling solvent, the SVA was performed at a significantly high temperature T=150 °C, much higher than what typical in SVA. The authors termed this process solvent vapour thermal annealing (SVTA). Must be underlined, though that, while temperature should be varied during SVA to thermal and solvent effects can be and surely are combined in every SVA experiment, still, the two effects are so strongly coupled, that in most scenarios, the selection of solvent processing conditions immediately imposes hard limits on the selection of temperature.

**Figure 9:**
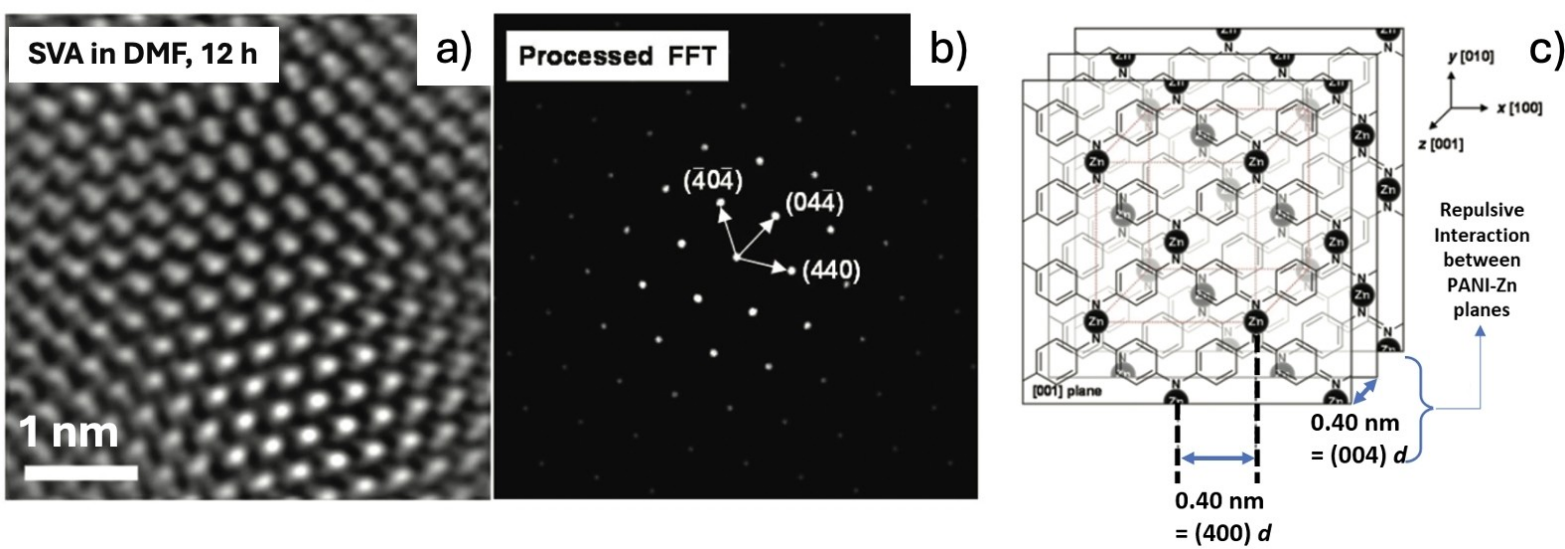
a) HRTEM and b) FFT images of PANI−Zn after SVA in DMF at 150 °C. c) Scheme of the proposed chemical arrangements of PANI−Zn. Figure adapted from Ref. [32].

Metal‐organic coordinated structures are typically produced by solvothermal reactions, but if this method is applied to a conducting polymer system, we lose the core advantages of polymer engineering. SVA of PANI blended with metal ions allowed instead *in situ* formation of coordination bonds in thin film states on a substrate. When using Zn ions, high‐resolution transmission electron microscopy (HRTEM) images revealed formation of an FCC crystal structure with regions of dark contrast in the HRTEM images due to Zn^2+^ coordinated with PANI, with a higher electron density around the metal ions than that in the organic atoms (Figure 9). The molecular crystal structure of PANI−Zn obtained by SVA was similar to that of a layered rock‐salt structure. Electric conductivity of the cross‐linked PANI−Zn reached 463 S m^−1^, 40 times higher than the one of a simple PANI−Zn mixtures.^[32]^

In SVA, the classical term ‘‘solution’’ cannot be used because, due to the nanometric thickness of the film, the number of solvent and solute molecules existing per unit area is comparable and they are confined in a very thin yet laterally extended layer. Interactions between the substrate, solute, and solvent are primarily of van der Waals type acting at molecular scale. For this reason, it is important to underline that the temperature effect in TESVA is markedly different from conventional thermal annealing. While in thermal annealing the temperature must be increased to get higher mobility, in TESVA the temperature must be reduced to have larger amounts of solvent on the surface, solubilize the molecules, and thus reduce the barrier to crystallization. Unlike vacuum evaporation, in TESVA the mobility of molecule at surfaces is not thermally activated, but rather it is promoted at lower temperatures. Under these conditions, a nanometric layer of liquid can facilitate the growth of macroscopic structures. If, however, ΔT becomes too negative, a macroscopic layer of solvent absorbs on the sample surface, and the system becomes similar to a classic bulk solution.

Even by controlling the temperature, typical SVA treatments can take very long times. However, by fast heating using microwaves, Zhang et al. demonstrated the possibility to perform fast SVA treatments, in few seconds. The anneal time was 60 or 180 s, while the temperature of the reaction vessel (as controlled by microwave intensity) could be varied.^[23]^ Tests performed with substrates able to adsorb microwaves with different intensities indicated an annealing mechanism in which both a solvent‐enriched environment and elevated substrate temperature played a role. Comparison with conventional thermal annealing showed that much shorter annealing times (several minutes vs 36 h) and lower temperatures were enough to achieve order.

## Evolution Towards Specialized Setups and Geometries

A major advantage of SVA is that it can be performed with a very simple setup, basically a simple sealed chamber, as mentioned above. This said, more elaborate setups have also been demonstrated, providing additional versatility and control on the coating morphology.

SVA can be performed right after solution deposition, without exposing the sample to dry air; in this way the dewetting processes which usually take place during the solvent evaporation phase right after spin coating and drop casting are greatly reduced, leading to more ordered structures. This approach is often pursued in simple drop casting or dip coating, by applying a cover glass on the sample right after the deposition, to slow down the solvent evaporation. In the last years, the process of removing the sample from solution, and perform a slow removal of the solvent has also been achieved in a more controllable, automated manner.

Several works have used SVA to assemble novel structures of the electron‐acceptor [6,6]‐phenyl C61 butyric acid methyl ester molecule (PCBM), which is one of the most used electron‐acceptor in organic photovoltaic. In 2010, Dabirian et al. demonstrated a simple setup to form highly crystalline and macroscopic PCBM architectures on solid surfaces was reported.^[33]^ In this setup the substrate was mounted on a computer‐ controlled arm which enabled to extract the sample from the solution at a highly‐controlled and very low speed. The vial was sealed and filled with a saturated atmosphere of solvent vapours, so that very slow solvent evaporation was attained, much slower and more controlled of what is usually obtained by drop casting in a closed atmosphere. Such a system can be built using very cheap hardware; in this case a programmable stepper from the famous LEGO Mindstorm© toy was used.^[34]^

In this way PCBM could be assembled in highly ordered, hexagonal shaped crystals ranging between 1–80 μm in diameter and from 20–500 nm in thickness, on a wide variety of surfaces such as SiO_x_, silanized SiO_x_, Au, graphite, amorphous carbon–copper grids and ITO.

Such automatized dip coating was then studied systematically in 2020 by Zhang et al., who performed a systematic study varying the extraction speed and angle between the sample and the liquid surface, combining experiments and simulation to better understand the role of contact angle, solvent evaporation and meniscus geometry on the final morphology of BTBT molecules, that were used as a test system.^[35]^

**Figure 10:**
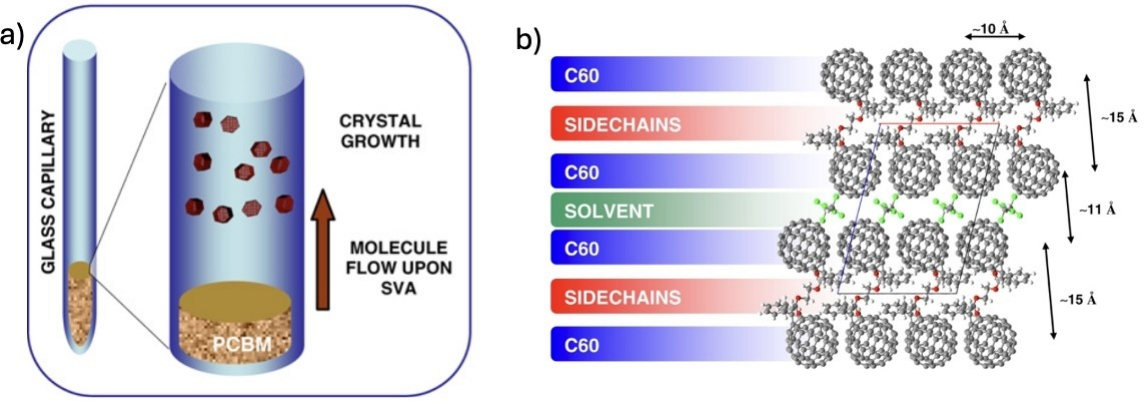
a) Cartoon illustrating the apparatus used and the SVA‐induced molecular transport along the capillary. b) PCBM molecular packing obtained by SVA, view along the b‐axis. The stated values relate to the minimum center‐to‐center distances in space for C60. Figure adapted from Ref. [36].

SVA could be performed even in geometries more complex than a simple flat substrate, as example inside thin glass capillaries for XRD, allowing to perform straightforward *in situ* analysis on the sample before and after SVA.^[36]^ In this way, large quantities of micro‐crystals of PCBM (Figure 10) were grown directly from PCBM powders previously deposited into glass capillaries.

PCBM aggregates were first suspended in chloroform, then transferred into the capillaries and then exposed for several days to SVA in chloroform vapour. Upon SVA, the previously amorphous PCBM powder evolved into micro‐crystals of PCBM with a size of tens of microns and a thickness below 500 nm, deposited on the sidewalls of the capillary. Given the large size of the original aggregates, reorganization during SVA was much slower than in previous cases, where the starting morphology was a thin, amorphous layer. That's why in this case, days were needed, instead of minutes, to grow ordered structures.

Interestingly, the best and most regular crystals were obtained at some millimeters’ distance from the original powder reservoir, above the initial level of the deposited material, where no PCBM was present at first and where a small flow of molecules could diffuse to create ordered crystals from scratch, demonstrating the long‐range nature of molecular diffusion during SVA. Macroscopic material transport was so strong that visible PCBM aggregates grew even at the top opening of the capillary, several centimeters far from the initial powder aggregates.

Noteworthy, this technique allowed to obtain a new crystalline phase, where the PCBM moieties are arranged in parallel layers (Figure 10b). This layered structure was characterized by the periodic arrangement of pairs of PCBM layers separated by solvent molecules and sandwiched between layers made of the side‐chains moieties of the fullerenes. The packing showed a peculiar, strong anisotropy in the minimum C_60_ center‐to‐ center distances: (i)≈10 Å for fullerenes belonging to the same C_60_ layer; (ii)≈11 Å for fullerenes within a primitive cell, separated by a solvent layer; and (iii)≈15 Å for fullerenes belonging to adjacent cells, separated by a side‐chain layer.

**Figure 11:**
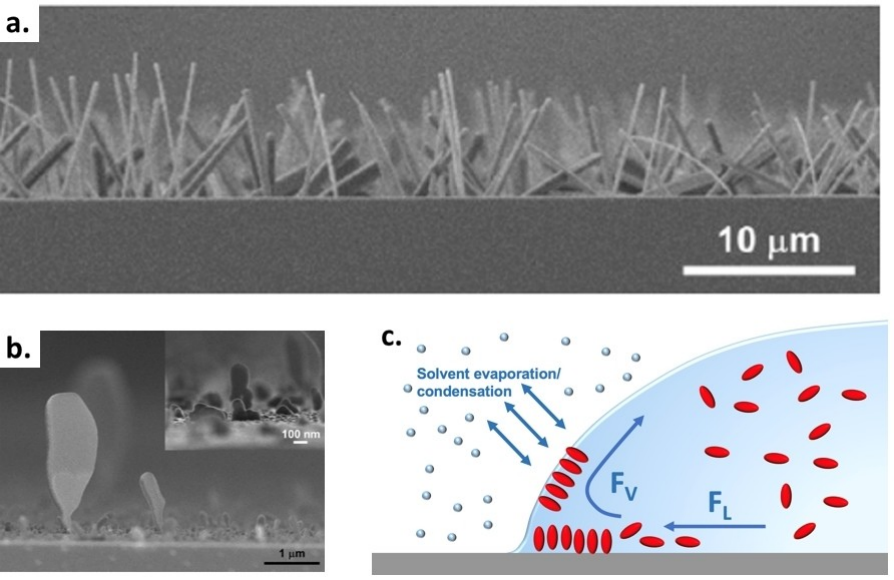
SEM images showing growth of C_60_ in vertical structures using SVA; a) nanowires and b) flat disks. Figures adapted from Ref. [37]. c) Proposed mechanism to explain vertical growth. Adapted from Ref. [1, 37].

Vertical growth of C_60_ in the form of wires and 2D disks was obtained by Kim et al. in 2013 combining SVA and droplet dynamics (Figure 11a and b).^[37]^ To this aim, they exposed thin films of C_60_, obtained by thermal evaporation, to a high vapour pressure of solvents like 1,3‐dichlorobenzene, mesitylene, m‐xylene and carbon tetrachloride (CCl_4_). In this way they obtained vertical growth of nanowires and disks with a length up to 7 μm. The vertical growth could be triggered by controlling fluid flow in solvent droplets formed on the surface. During the drying of a droplet of solution, the edges of a droplet are often pinned on defects of the surface; then, a lateral flow F_L_ moves from the center to the edges of the droplet to replenish solvents to the perimeter. If F_L_ is strong enough, solutes accumulate at the edges of the droplet causing the typical “coffee ring effect”. Besides F_L_, an additional flow F_V_ can be present moving along the walls of the droplet, due to thermal and concentration gradients (e. g. Marangoni flow) (Figure 11c).^[38]^ According to the authors, SVA greatly reduced the movement speed of the droplet boundary maximizing vertical drying force of the solvent (F_V_), thus favouring vertical crystallization of C_60_. Also in this case, solvent molecules were included in the crystalline structure of the vertically grown C_60_ nanowires.

Self‐assembling with SVA can be performed not only on a surface but on the pristine solution itself, exposing it to vapours of an anti‐solvent which diffuse in the solution, reducing the solubility of eventual molecules and driving self‐assembly in liquids. In 2018 Zhao et al.^[2]^ assembled microwires of unfunctionalized C_60_ fullerene of length up to 4 mm, exposing solutions of C_60_ in m‐xylene (a good solvent) to vapours of methanol, ethanol or isopropanol, all bad solvents for the molecule. This kind of “solvent exchange” process, even if using a setup similar to SVA, is not the key argument of this review, so it is only mentioned here.

## Dynamic Control of SVA: Towards User‐Friendly Systems

The typical SVA setup can be very simple, usually the samples are placed in a chamber with a reservoir of liquid solvent(s), whose evaporation provides a saturated atmosphere. The use of solvent mixtures (versus a single solvent) offers an additional handle for tuning solvent selectivity and provides a way to achieve the desired morphology. However, in the context of SVA under a static atmosphere, controlling the partial vapour pressures of different solvents can be a challenge.

Several groups demonstrated the possibility to perform SVA in continuous flow systems, exposing the sample to a saturated vapour stream produced by bubbling inert gas through a liquid solvent, measuring its amount using mass‐flow controllers (MFCs). This enables to control the vapour concentration and has been used, for example, to create phase diagrams of the morphologies produced by SVA in block copolymers.^[21]^

**Figure 12:**
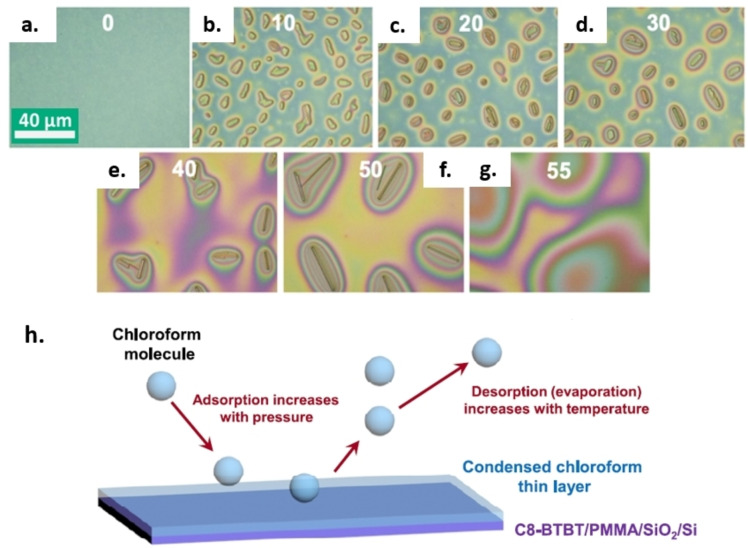
a–g) OM images from a video of C8‐BTBT rod growth at 28 °C and 220 Torr in solvent gas. h) A schematic of the dynamics of chloroform adsorption and desorption. Figures adapted from Ref. [31].

In 2020, Yu et al. described the growth mechanism and coarsening kinetics of yet another an organic semiconductor, the BTBT derivative benzothieno[3,2‐b][1]benzothiophene, on PMMA films. They performed SVA in chloroform varying systematically the substrate temperature in the range 25–32 °C and the solvent gas pressure in the range 160–240 Torr. The SVA progress was studied using optical microscopy (Figure 12a–g), XRD and Raman spectroscopy.^[31]^

They proposed a simple model where the adsorption and desorption of solvent molecules on the film are dynamically competitive. The amount of chloroform adsorbed on the film increased with increasing the gas pressure and decreased with increasing the temperature, since the higher temperatures enhance the evaporation of chloroform molecules (Figure 12h). Initially, chloroform drops formed and promoted the assembly of small rods of BTBT, each of them trapped in an individual drop. When the pressure was high enough the drops coarsened, and the rods that at this point were connected by liquid solvent could interact and exchange material. The smaller rods decreased in size by transferring their molecules to the larger rods and by partial melting into the liquid. Noteworthy, rods interacted with each other only when they were in a liquid chloroform drop formed by SVA. Finally, if the vapour pressure was too high, the system became comparable to a bulk solution, as mentioned previously, and the rods dissolved completely in liquid chloroform. By measuring the total rod volume using ex‐situ AFM, Yu et al. could find the ideal SVA conditions to produce BTBT rods of length >50 μm, that were successively used to realize transistor devices, showing high mobilities up to 7.67 cm^2^ V^−1^ s^−1^.

**Figure 13:**
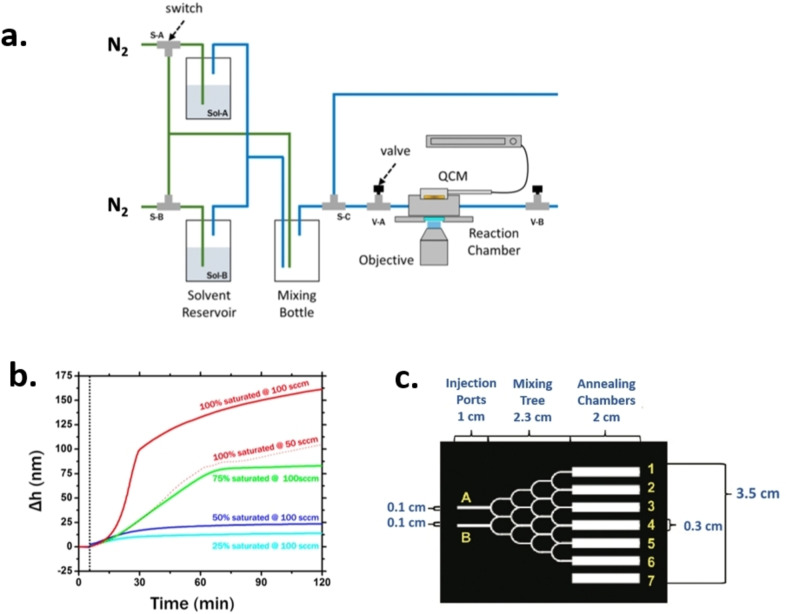
a) Solvent vapour delivery system schematic diagram. MFCs regulate the carrier gas flow via the sample chamber and the proper solvent reservoirs. Throughout, switches and valves are used to direct and regulate flow. Figure adapted from Ref. [10]. b) (Solid lines) Change in film thickness (Δh) over time at different solvent partial vapour pressures obtained through dilution from saturation. The data displayed pertain to a total flow rate of 100 sccm (Q_nit_). Change in film thickness with time at saturated toluene vapour pressure at a total flow rate of Q_nit_=50 sccm is shown by the dot‐plot line. The temperature was maintained at 21 °C for 5 minutes (vertical dashed line) in both sets of data. Figure adapted from Ref. [10]. c) Design masks for a solvent annealing device. Solvent vapours enter the final device via injection ports A and B, mix in the mixing tree, and then go through annealing chambers 1 through 6. Chamber 7, which functions as a control chamber in the solvent annealing device, is separated from the mixing tree. Figure adapted from Ref. [22].

In 2016, Hoang et al. built a SVA setup able to monitor *in‐situ* the condensation of solvent on a polymer layer, and the increase of diffusivity in the layer due to SVA.^[10]^ To do this, they performed SVA on two similar samples of poly(methyl methacrylate) (PMMA), one positioned on a quartz crystal microbalance (QCMs), the other deposited on a cover slip for fluorescence measurement (Figure 13a). Vapourized solvent was dosed in a controlled fashion using a series of MFC in nitrogen flow. The measurements allowed to control and measure the amount of solvent that was adsorbed by the polymer via swelling; the solvent thickness was tuned from 0 nm up to ≈175 nm by varying the saturation degree of the solvent vapours (Figure 13b). This allowed also to measure the diffusion coefficient of small quantum dots of CdSe/ZnS with a diameter 3 nm, and to monitor in real time the formation of fluorescent aggregates of phenylenevinylene (PPV) by SVA of a PPV‐PMMA blend. The diffusion and aggregation of PPV polymer chains in PMMA followed the Ostwald ripening model, in which single smaller aggregates were losing polymer chains which then re‐dissolved and incorporated into larger aggregates.

Besides the active control of solvent vapour using gas valves, also passive microfluidic devices could be used to produce discrete gradients in solvent vapour composition and/or concentration. In 2011, Albert et al. produced a “mixing tree” to obtain a gradient in composition in solvent vapours (Figure 13c). The design masks incorporated two “injection ports” for solvent entry into the device, and seven “annealing chambers”. An additional control chamber not connected to the mixing tree was as blank. The goal of this device was to enable faster and more robust exploration of SVA parameter space, providing insight into self‐assembly processes.^[22]^

**Figure 14:**
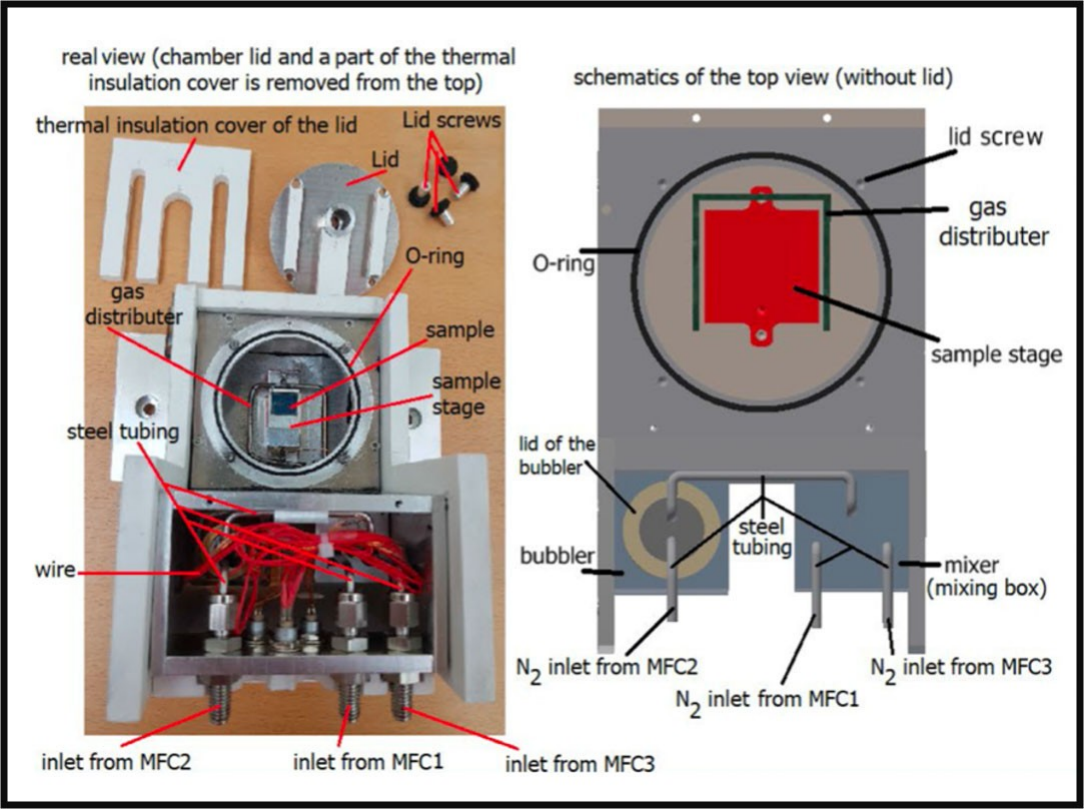
The top view of the chamber after removing the lid showing the sample stage, gas distributor, gas inlets from mass flow controllers, and a thin film sample. Figure adapted from Ref. [18].

Being a fast technique that can be performed also in closed SVA chambers, GISAXS at synchrotron sources is an ideal technique to study *in operando* the SVA process, providing information on the self‐assembled structures both laterally and along the film normal. In 2017, Posselt et al. published an extensive study combining theoretical studies and experimental techniques to manipulate the structures of polymer layers, and follow their evolution upon SVA in real time.^[16]^ In 2023, Ariaee et al.^[18]^ developed a compact setup for SVA, weighting ≈2 Kg and easy to transport (Figure 14). This allowed to perform SVA, for example, at synchrotron beamlines for small‐angle X‐ray scattering (SAXS) or GISAXS studies. This setup had windows to allow the penetration of X‐rays and a tool to better distribute the gas on the sample surface. Solvent concentration in the exhaust gas was continuously monitored by UV‐absorption. Thanks to the compact setup, the atmosphere could be changed from dry (100 % N_2_ flow) to wet (i. e., 0 %–100 % solvent humidity) and vice versa within only few minutes.

## Conclusions and Perspectives

In the intricate field of molecular deposition techniques, the solvent‐vapour‐annealing related methods are certainly among the sharpest tools available in the chemist instrument box, when sitting at the famous “watchmaker's table” mentioned in the introduction. We shall compare the SVA process with the most common deposition techniques as schematized in Table 2, highlighting the many advantages of SVA. We already mentioned about the limitations of drop casting and spin coating. The former often leads to non‐uniform coatings and defects (e. g. “coffee stains”) and is slow. The latter is fast and uniform, but a significant amount of the material is wasted, it requires a complex spin coater machine and, most importantly, any surface self‐assembly need to be achieved in the few seconds during which the solvent evaporates. In both cases the size of the cast drop or of the spin coater machine limit the possible size of the coating, that's why these techniques are mainly used in electronics industry, were wafer sample size is on the centimetres scale.

**Table 2:** Schematic comparison of different common deposition techniques.

	DROP CASTING	SPIN COATING	DIP COATING	SPRAY COATING	SVA
Number of processing steps	1	1	1	1	2
Cost	++	−−	−−	−−	+
Speed	−	+	−	++	−
Uniformity	−	+	−	+	+
Material waste	+	−−	+	−	++
Compatible with large samples	−−	−	−	++	+
Compatible with slow self‐assembly kinetics	+	−	−	−	++
Compatible with poor solvents	−	−	−	−	++

Other widespread techniques like dip coating or spray coating overcome these issues and are commonly used at industrial level also for large (sq. meters) coatings, but they also have issues; dip coating requires a good control of sample movement and solvents with excellent wetting, while spray coating requires complex equipment, and fast self‐assembly times.

Most importantly, all conventional techniques require the molecules to be processed in an excellent solvent, which is not always the solvent ideal to achieve the desired morphology or, in case of materials for electronics, the best performance. By decoupling the deposition and the self‐assembly steps, SVA overcomes this problem providing to the researchers a much greater versatility than any other processing technique.

The essence of the SVA process resides in the orders of magnitude increase of the molecular diffusivity on the surface, thanks to the presence of the solvent molecules surrounding and mobilizing the target ones, in a manner similar to what lubricants typical do, but at the molecular scale. This increased mobility allows molecules to rearrange over extremely long distance, realizing structures with a high degree of morphological order also at macroscopic scales. The process can be realized with unexpensive equipment and has practically no intrinsic limitations on the maximum scales at which it can be applied. Moreover, the analogy between the solvent molecules and lubricants suggests the intriguing possibility that mastering all the microscopic details of the vapour annealing processes can have an impact not only in the field of material growth and related properties, but also on very distant topics, such as mechanics and energy.

Novel “clever tricks” are constantly being invented to extend the capabilities of the SVA‐related approaches and to improve the degree of control of the various parameters involved. In the simplest SVA processes, the possibility to change the type of solvent varying between polar/apolar, high/low boiling point, high/low solubility for a specific molecular type or even realizing mixture with more than one solvent type, already offer a high degree of tunability to achieve the desired molecular film morphology. In TESVA, the additional control on the temperature difference between the sample and the solvent reservoir is a further important parameter to gain control on the actual quantity of solvent on the sample and hence of the microscopic processes. The aforementioned points have been exploited to showcase the creation of novel, highly ordered structures that were not achievable through alternative deposition techniques. This has generally resulted in a significant enhancement of the initial molecular materials’ characteristics, in particular concerning their electrical charge mobility.

More recently, the precise control on the vapours flow and pressure and the realization of user‐friendly, compact, transportable systems than can be easily coupled with sophisticated characterization techniques, have enabled SVA methods to provide a level of control over microscopic process details so refined that is now approaching the precision typically reserved for ultrahigh vacuum based techniques, in which the control over the deposition details extends down the molecular scale.

While the use of SVA has advantages related to the better control and tunability, it has also significant drawbacks, that will limit its use at industrial level. The addition of a post‐processing step after deposition increases the complexity of the industrial coating process, and can thus often be considered not applicable in many industrial sectors. Besides the number of steps, SVA often requires several hours of annealing, and also this is a significant obstacle for large‐scale applications. However, SVA can be applied in parallel to large areas of samples, depending only on the size of the SVA chamber, and could thus be compared to other slow processes already performed in the industry on the hours timescale; we do not exclude that it could be applied on large scale for post‐processing treatments of high‐value products, similar to what currently done, as example for curing of carbon fiber composites in autoclaves.

All the original works described in this review, along with numerous others present in the literature, demonstrate that SVA and all the other related techniques can no longer be regarded as simple post‐processing methods, but as a proper way to grow novel molecular materials. Indeed, while most of the initial reports were focused on improving the properties of already existing material by enhancing the molecular order, more and more often SVA is used to obtain new molecular order and arrangements, characterized by different physical and chemical properties, that cannot otherwise be realized with more traditional methods.

## Conflict of Interests

The authors declare no conflict of interest.

## Biographical Information

*Vasiliki Benekou received her bachelor's degree in Physics from the University of Patras in 2016. Later, she worked as a Research assistant at FORTH/ICE‐HT Institute in Patras, Greece and completed an M.Sc. in Materials Science at the University of Patras. In 2020, she was awarded an ITN Marie Curie Fellowship to pursue her PhD at CNR Institute for Organic Synthesis and Photoreactivity (ISOF) in Bologna, Italy and the University of Modena and Reggio Emilia. She works on the synthesis and characterization of synthetic 2D materials with a focus on their electronic properties under the supervision of Dr. Vincenzo Palermo*.

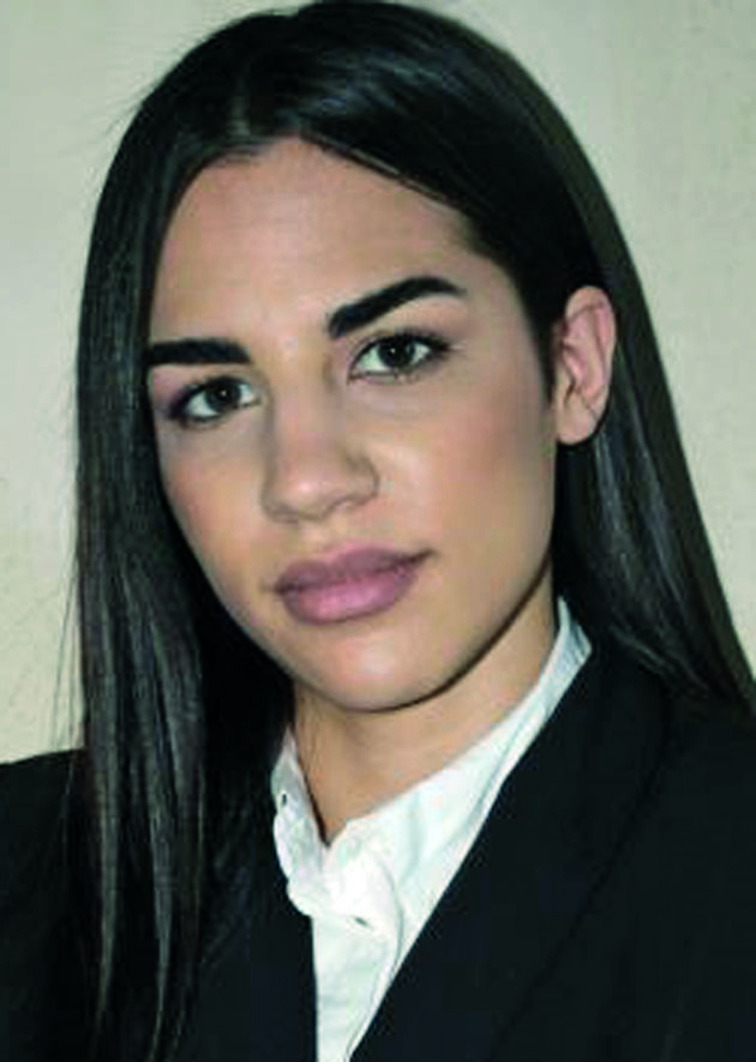

## Biographical Information

*Andrea Candini is senior researcher at the CNR Institute for Organic Synthesis and Photoreactivity (ISOF) in Bologna, Italy. He received his PhD in 2007 in physics from the University of Modena and Reggio Emilia, studying molecular nanomagnets and magnetic devices. He then had post‐doctoral positions at CNRS Grenoble (France) and CNR in Modena working on molecular quantum spintronics and graphene nanodevices before moving to ISOF in Bologna in 2017. His current research interests include the study of low dimensional materials on surfaces via scanning probes and characterization and engineering of novel materials for quantum sensing, in particular in biological systems*.

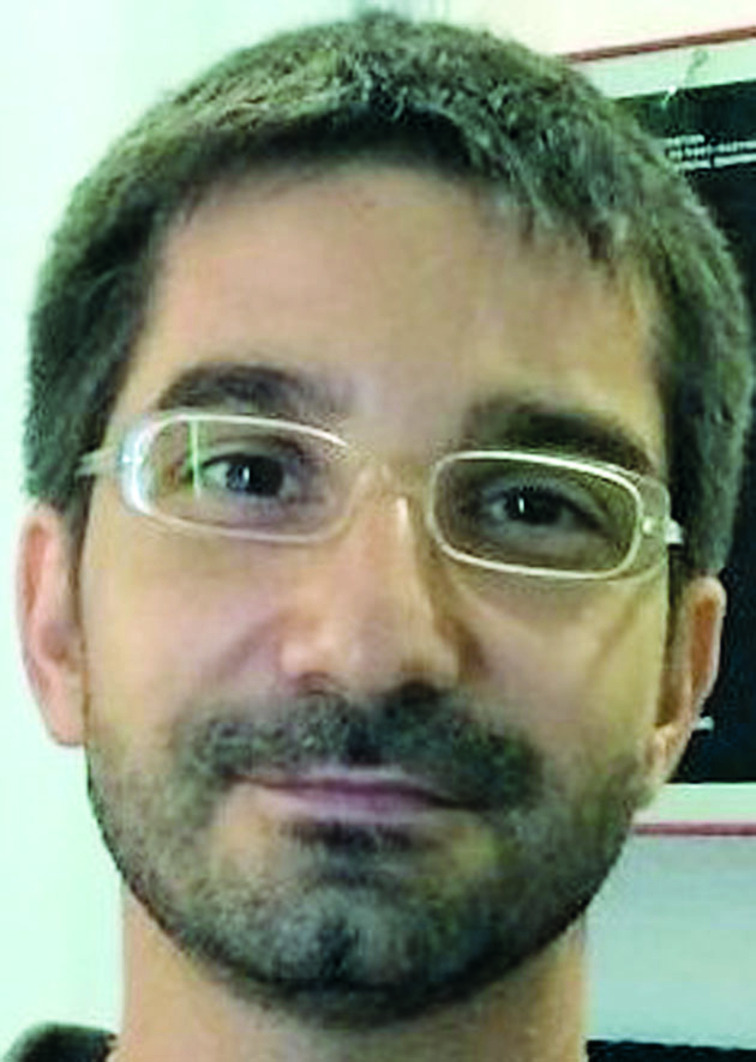

## Biographical Information

*Andrea Liscio received his PhD in 2003 at the Roma Tre University, working on coincidence electron spectroscopies applied to low‐dimensional systems. Since 2004, he focused his scientific activities on Scanning Probe Microscopy beyond imaging, developing general tool for quantitative analysis to correlate morphology and the electronic properties at nanoscale on 2D materials. Currently he is Senior Researcher at CNR‐IMM, his current research interests include the development of up‐scalable approaches for production and characterization of solution‐processed graphene and related 2D materials for large‐area electronics, water treatment and aerospace application*.

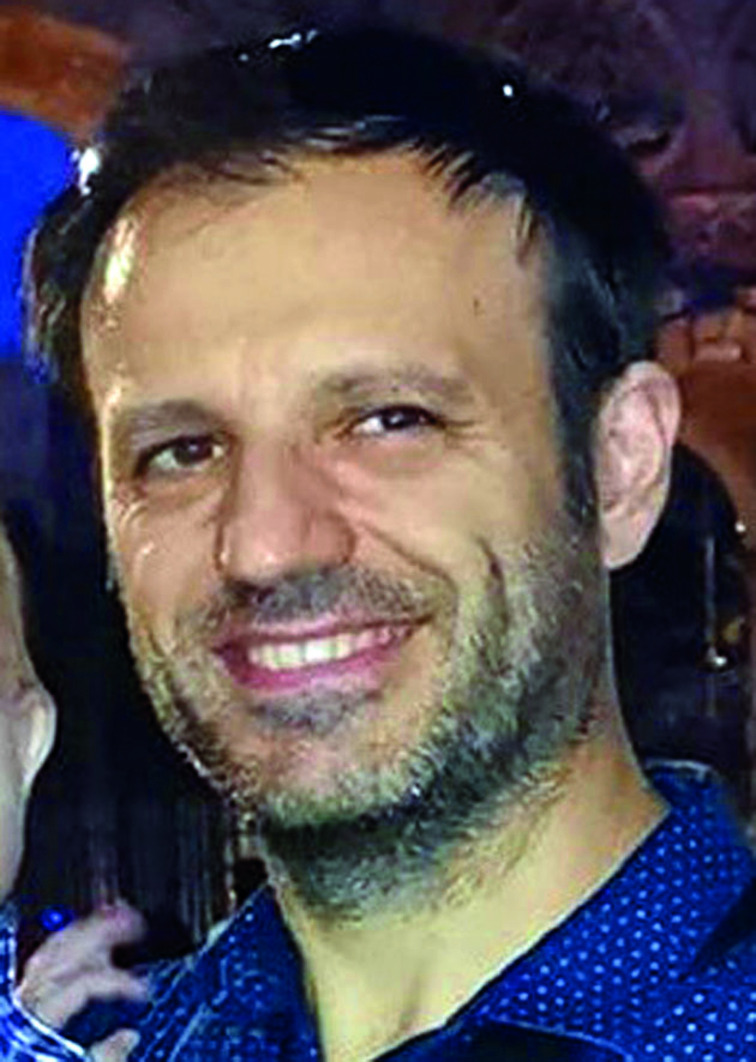

## Biographical Information

*Vincenzo Palermo is the director of the CNR Institute for Organic Synthesis and Photoreactivity (ISOF) in Bologna, Italy, and associated professor of Chalmers University (Sweden). He uses nanotechnology and chemistry to create new materials for electronics, aerospace and biomedical applications. His work has been published in >200 scientific articles on international journals in chemistry, nanotechnology and materials science (>11000 citations, h‐index 53), many of them in collaboration with key industrial partners in Europe (Airbus, BASF, Stellantis, Leonardo etc.). His research interests include supramolecular chemistry, energy storage, composites, water purification and biosensors*.